# Assessment of Nursing Shortage and Calculation Methods in Saudi Arabia: A Cross-Sectional Study in Government Hospitals

**DOI:** 10.7759/cureus.71339

**Published:** 2024-10-12

**Authors:** Adel A Alhindi, Ilias Mahmud, Hassan Altakroni, Yousif M Elmossad, Md Dilshad Manzar, Majed Alamari, Khalid Alhusseinan

**Affiliations:** 1 Nursing Administration, Al-Qassim Health Cluster, Buraidah, SAU; 2 Epidemiology and Public Health, College of Public Health and Health Informatics, Qassim University, Bukayriah, SAU; 3 Nursing Office, College of Applied Medical Sciences, Buraydah Colleges, Buraidah, SAU; 4 Public Health, King Faisal University, College of Applied Medical Sciences, Alhasa, SAU; 5 Nursing, College of Applied Medical Sciences, Majmaah University, Majmaah, SAU; 6 Nursing, Hafer Al-batin University, Riyadh, SAU; 7 Human Resources, Al-Qassim Health Cluster, Buraidah, SAU

**Keywords:** estimation methods, evidence-based practices, government hospitals, healthcare workforce planning, nursing directors, nursing shortage, nursing staffing, patient acuity, professional judgment, saudi arabia

## Abstract

Background

This cross-sectional study aimed to explore the prevalence of nursing staff shortages in government hospitals in Saudi Arabia, investigate the methods used to assess these shortages, and examine the association between the demographic variables of nursing directors and their ability to calculate staffing shortages.

Methods

A survey was conducted among current and past nursing directors in government hospitals across Saudi Arabia. Data were collected using a semi-structured questionnaire that covered demographic information, work-related characteristics, and perceptions of nursing staffing plan estimation methods. Statistical analyses, including descriptive statistics, chi-squared tests, and logistic regression, were performed to analyze the data.

Results

A total of 279 nursing directors participated in the study, with the majority being female (156, 55.9%) and Saudi nationals (220, 78.9%). Most participants held a Bachelor of Nursing degree (161 participants, 57.7%) and had over 10 years of professional nursing experience (68.4%). Most respondents recognized a shortage of nursing staff in their hospitals (222, 79.6%), with 192 participants (68.8%) reporting fewer than 278 nurses in their facilities. Various methods were used to estimate nursing staffing plans, with the professional judgment approach being the most preferred answer by 128 participants (45.9%). Memos, training courses, and internet browsing were the primary sources of information on calculation methods. While no significant association was found between nursing staff shortages and estimation methods, there was a significant association between nursing education level and the approach used for estimation.

Conclusion

Standardized, evidence-based approaches to workforce planning are crucial for effectively addressing nursing staff shortages in Saudi Arabian government hospitals. Education and training initiatives for nursing leaders are essential to ensure accurate estimation of staffing needs and improve the overall quality of healthcare services.

## Introduction

Healthcare administrators, particularly nursing leaders, face critical decisions regarding nursing team size and composition. Calculating nursing staffing plans involves determining the optimal number and mix of nursing staff required to provide safe, effective, and efficient patient care. Several methods can be used for this purpose, depending on patient acuity, hospital size, and available resources [1]. In calculating nursing staffing plans, several common approaches are employed to ensure optimal patient care and efficient resource allocation. One such method is professional judgment, which draws upon the expertise and experience of nursing leaders to estimate staffing needs. These estimates are based on various factors, including patient acuity, case complexity, and unit workload [2].

Another approach is the nurse per occupied bed (NPOB) method, which determines staffing levels based on the number of patients occupying beds in the hospital or unit. While this method considers patient volume, it may not consider variations in patient acuity or nursing workload [3]. A more nuanced approach is acuity-adjusted staffing, which adjusts staffing levels based on patient acuity levels, reflecting the severity of illness or care needs. This method tailors nursing staff requirements according to the total acuity score of patients on the unit [4].

The timed-task/activity-based approach is another strategy that involves breaking down nursing tasks and activities into standardized time intervals. Staffing needs are then estimated based on the time required to complete each task [5]. Regression-based models represent a data-driven approach, utilizing statistical regression analysis to identify factors influencing nursing workload and staffing requirements. These models analyze variables such as patient census, patient acuity, and nurse-patient ratios to develop predictive models that can estimate staffing needs based on observed patterns and trends [6].

Lastly, some organizations opt for combination methods, integrating various factors such as patient acuity, workload intensity, and unit-specific characteristics to determine staffing levels [7].

When calculating nursing staffing plans, it's essential to involve nursing leaders, frontline staff, and other stakeholders to ensure that staffing levels align with patient care needs and organizational goals [8]. Regular monitoring and adjustment of staffing plans based on ongoing assessment of patient acuity, workload, and staff feedback are also critical to maintaining optimal staffing levels and ensuring high-quality care delivery [4].

Addressing the global shortage of registered nurses necessitates competent nurse leaders equipped with managerial skills and knowledge of staffing calculation methods. In Saudi Arabia, despite its economic strength, the shortage of healthcare professionals persists, posing risks to public health [9]. Contributing factors include an aging population, workforce, gender inequality, high turnover, and inadequate staffing plans [10]. Insufficient staffing hinders patient care, highlighting the need for comprehensive strategies to address nursing shortages [11].

This study aimed to investigate the nursing staff shortage in Saudi Arabian government hospitals and the methods used to assess this shortage. Additionally, it explored the sources of knowledge among staff nurses regarding different calculation methods and examined the association between staff nurses' demographic variables and their ability to assess nursing staff shortages independently. The findings of this study may contribute to improving healthcare quality by increasing awareness of effective approaches for addressing nursing shortages through accurate workforce measurements and optimal distribution of nursing staff across hospital departments.

## Materials and methods

A cross-sectional survey was conducted targeting current and past nursing directors in government hospitals across all regions of Saudi Arabia. The research focused on assessing nurse leaders' knowledge of nursing staff distribution within hospital units using standard calculation methods for estimating staffing plans.

Using Epi InfoTM, a sample size of 164 was determined. Data were collected via a semi-structured questionnaire in English and Arabic, covering demographic and work-related characteristics, as well as perceptions of nursing staffing estimation methods. The validity and reliability of the questionnaire were ensured through expert evaluation and statistical analysis. Descriptive and inferential statistics were used for data analysis, including frequency distributions, Chi-squared tests, and logistic regression.

The study employed a cross-sectional survey design targeting current and past nursing directors in government hospitals across all regions of Saudi Arabia. The research focused on assessing nurse leaders' knowledge of nursing staff distribution within hospital units using standard calculation methods for estimating staffing plans.

Invitations to participate were extended to all government hospitals in Saudi Arabia. A total of 284 nurses completed the survey, with 5 responses excluded due to incomplete data.

Data collection involved administering a semi-structured questionnaire tailored for this study, available in both English and Arabic languages to accommodate participants' linguistic preferences. The questionnaire comprised two sections:

Section one encompassed 13 questions, focusing on demographic information (e.g., gender, nationality, educational level) in questions 1 to 5 and work-related characteristics (e.g., hospital bed capacity, occupancy rate) in questions 6 to 13. Section two comprised eight closed-ended questions with binary response options ("Yes" or "No"), aimed at gauging respondents' perceptions or knowledge regarding estimating nursing staffing plans.

To ensure the validity of the questionnaire, a Likert scale research instrument evaluation parameter tool was utilized. This tool was assessed by a panel of three experts to evaluate its face and content validity, ensuring correctness, clarity, and organization of the questionnaire, along with accurate translation into Arabic.

After ensuring the validity of the research tool, its reliability was tested on 31 nursing directors from the Ministry of Health Hospitals. Reliability was assessed using McDonald's Omega and Greatest Lower Bound (GLB) methods, yielding excellent results with a GLB of 0.97 and good reliability with McDonald's Omega of 0.78.

Data analysis was conducted using the IBM Corp. Released 2015. IBM SPSS Statistics for Windows, Version 23.0. Armonk, NY: IBM Corp. Descriptive analyses were performed to report the frequency and percentage of study variables, presented in tables and figures.

Chi-squared tests were employed to investigate the association between the approach used to calculate nursing staff shortages and the level of education of staff nurses. Multivariable logistic regression analyses were conducted to explore sociodemographic factors associated with estimating nursing staff shortages. Additionally, multivariable logistic regression was used to examine the association between nurse directors' socio-demographic characteristics and their exposure and attitudes toward standard approaches for estimating nursing staffing plans.

## Results

A total of 279 current or past nursing directors participated in the study, with (156, 55.9%) being female and (220, 78.9%) being Saudi nationals. Table 1 describes the socio-demographic characteristics of the study participants. More than half (57.7%) held a Bachelor of Nursing degree, and most participants, 191 (68.4%), had over 10 years of professional nursing experience. However, as nursing directors, the majority, 173 (62%), had 1 to 3 years of experience.

Regarding work-related characteristics, 68.8% of the nurses mentioned having less than 278 nurses in their hospitals, with 79.6% recognizing a shortage of nursing staff. Among the nursing directors surveyed, 46.2% worked in hospitals with a bed capacity of less than one hundred beds, while 25.8% worked in hospitals with a bed capacity ranging from 101 to 300 beds (Table 1).

**Table 1:** The socio-demographic characteristics of the study participants

Variables		Frequency	Percent
Gender	Male	123	44.1
	Female	156	55.9
Nationality	Saudi	220	78.9
	Non-Saudi	59	21.1
Education	Nursing diploma	77	27.6
	Bachelor of Nursing	161	57.7
	Postgraduate in nursing	41	14.7
Years of experience as a nurse	1-3 Year	20	7.2
	4-6 Year	29	10.4
	7-9 Year	39	14
	10-12 Year	50	17.9
	13-15 Year	65	23.3
	16 Years and above	76	27.2
Years of experience as a Nursing Director	1-3 Year	173	62
	4-6 Year	46	16.5
	7-9 Year	28	10
	10-12 Year	18	6.5
	13-15 Year	8	2.9
	16 Years and above	6	2.2

Related to the bed occupancy rate in hospitals, results showed that 76 (27.2%) of the nurses mentioned a bed occupancy rate ranging from 26 to 50%, and 63 nurses (22.6%) reported rates from 51 to 75%, with the average bed occupancy rate being 94.5%. In terms of the standard used to estimate nursing staffing plans, more than half of the nurses preferred the professional judgment approach.

Regarding estimation methods, 128 nurses (45.9%) preferred the professional judgment approach, and over half used this method for estimating nursing staffing plans. Memos were the primary source of information (151, 54.1%) on calculation methods, followed by training courses (110, 39.4%) (Table 2).

**Table 2:** Hospital nursing staffing and resource allocation

	Variables	Frequency	Percent
Number of nurses in the hospital	Less than 278 nurse	192	68.8
	279 and more	87	31.2
Shortage of nursing staff in the hospital	Yes	222	79.6
	No	57	20.4
Hospital bed capacity	Less than 100 beds	129	46.2
	101 to 200 beds	72	25.8
	301 to 400 beds	42	15.1
	401 to 500 beds	19	6.8
	> 500 beds	17	6.1
The bed occupancy rate in hospital	0-25	32	11.5
	26-50	76	27.2
	51-75	63	22.6
	76-100	60	21.5
	>101	48	17.2
Preferred approach for estimating the nursing staffing plan	Professional judgment approach	128	45.9
	Acuity-quality method	13	4.7
	Nurses per occupied bed method	54	19.4
	Other methods	84	30.1
The approach used to estimate the nursing staffing plan	Professional judgment approach	145	52
	Nurses per occupied bed method	34	12.2
	Acuity-quality method	13	4.7
	No methods	87	31.2

**Figure 1:**
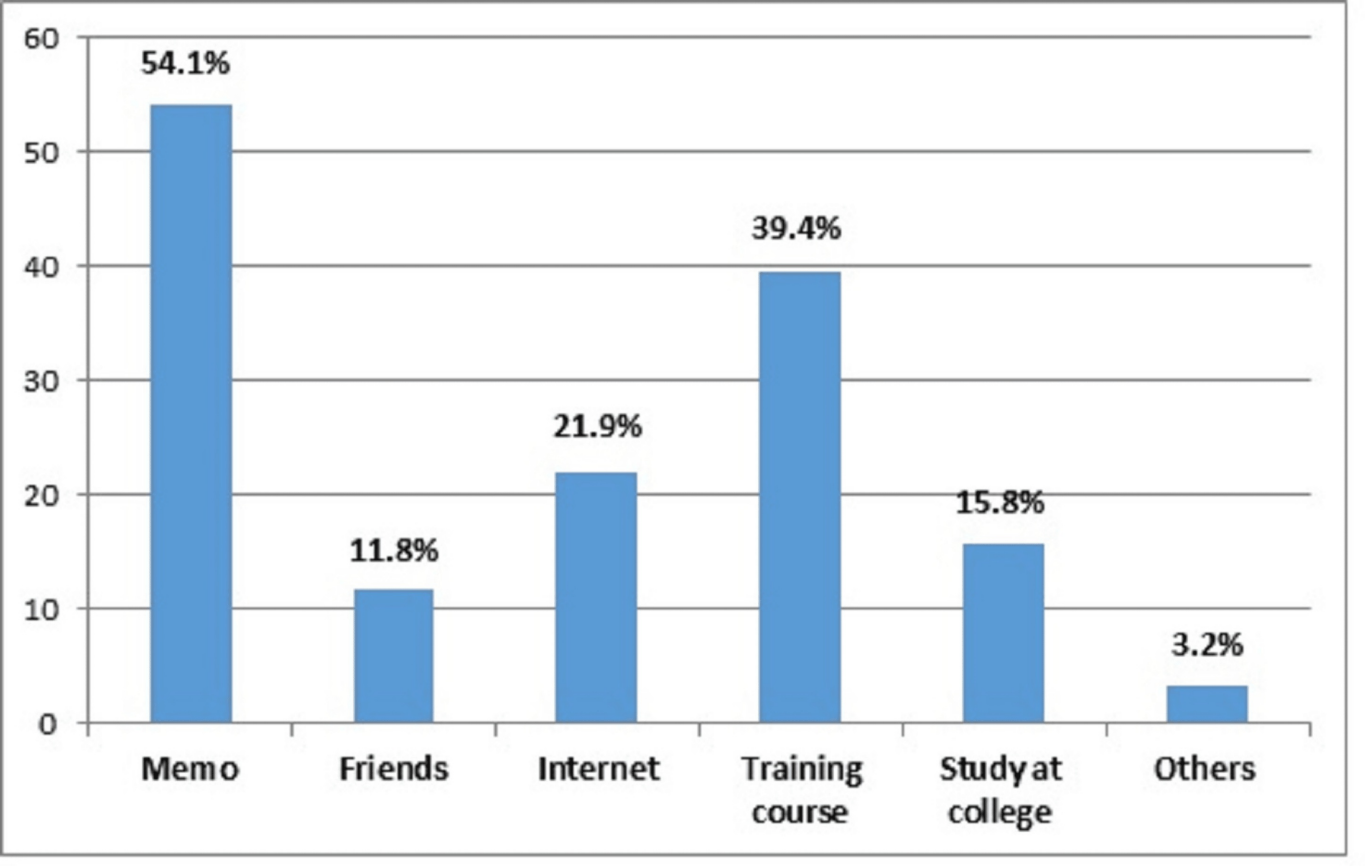
Source of information on calculation methods for estimating the nursing staffing plan

Figure 1 illustrates that memos were the most prevalent source of information on calculation methods for nurses (151, 54.1%), followed by training courses (110, 39.4%) and internet browsing (61, 21.9%).

Table 3 presents nursing directors’ exposure and perceptions of approaches to estimate nursing staffing plans. Most nurses identified a shortage of nurses in their hospitals (220, 79.2%), with the majority attending special courses on estimating nursing staffing plans after graduation (204, 73.1%). Moreover, 232 (83.2%) recognized differences in estimating the nursing staffing plan among hospital departments.

**Table 3:** Nursing staffing plan estimation practices

Variables	Yes	No
	n (%)	n (%)
Have you studied calculation methods for estimating the nursing staffing plan during your college period?	124 (44.4)	155 (55.6)
Is there a standard for estimating the nursing staffing plan in your hospital?	222 (79.6)	57 (20.4)
Is there a difference in estimating the nursing staffing plan among hospital departments?	232 (83.2)	47 (16.8)
Are customs and traditions obstacles to applying calculation methods in estimating the nursing staffing plan?	117 (41.9)	162 (58.1)
Have you received special courses about calculating methods of estimating the nursing staffing plan?	204 (73.1)	75 (26.9)
Is it possible to apply calculation methods for estimating the nursing staffing plan in your hospital?	232 (83.2)	47 (16.8)
Do you think that estimating nursing staffing plans in KSA hospitals should have a common standard or guidance?	259 (92.8)	20 (7.2)

Table 4 indicated no significant association between nursing staff shortage and estimation method (p = 0.259). However, nursing education level was significantly associated (p = 0.017) with the approach used. Female nurses perceived customs and traditions as more significant obstacles (p < 0.05), and nurses with higher education levels had more exposure to calculation methods (p < 0.05).

**Table 4:** Relationship between estimation approaches for nursing staffing plan, shortage in nursing staff, and level of education in nursing

The approach to estimate nursing staffing plan	Shortage in nursing staff			Level of education in nursing			
	No n (row %)	Yes n (row %)	p-value	Diploma n (col. %)	Bachelor n (col. %)	Postgraduate n (col. %)	p-value
Professional judgment approach	28 (19.3)	117 (80.7)	0.259	33 (42.9)	80 (49.7)	32 (78.0)	0.017
Nurses per occupied bed method	5 (14.7)	29 (85.3)		12 (15.6)	18 (11.2)	4 (9.8)	
Acuity-quality method	1 (7.7)	12 (92.3)		4 (5.2)	8 (5.0)	1 (2.4)	
No methods	23 (26.4)	64 (73.6)		28 (36.4)	55 (34.2)	4 (9.8)	

In Table 5, female nurses had a significantly higher perception that customs and traditions were considered an obstacle to applying calculation methods in estimating the nursing staffing plan (p < 0.05) compared to male nurses. Additionally, nurses with Bachelor of Nursing and postgraduate studies had a significantly higher declaration of studying calculation methods for estimating the nursing staffing plan in their college (p < 0.05). Moreover, nurses with 1-6 years of experience as the director had a significantly higher declaration that they did not receive special courses about calculating methods of estimating the nursing staffing plan (p < 0.05).

**Table 5:** Socio-demographic factors associated with perception of shortage in nursing staff in Saudi Arabia

Variables		Found a shortage in nursing staff. n (%)	p-value	Odds Ratio (95%Confidence Interval)
Gender	Male	103 (36.9)		
	Female	119 (42.7)	0.021	2.37 (1.14-4.91)
Nationality	Saudi	171(61.3)		
	Non-Saudi	51 (18.3)	0.016	0.31 (0.12-0.80)
Experiences as Nurse	1-3 years	13 (4.7)		
	4-6 years	24 (8.6)	0.339	0.54 (0.15-1.93)
	7-9 years	32 (11.5)	0.51	1.54 (0.42-5.61)
	10-12 years	42 (15.1)	0.867	1.10 (0.36-3.36)
	13-15 years	50 (17.9)	0.423	1.54 (0.54-4.43)
	16 years above	61 (21.9)	0.862	0.92 (0.38-2.24)
Experience as a nursing director	1-3 years	138 (49.5)		
	4-6 years	38 (13.6)	0.873	1.22 (0.11-13.34)
	7-9 years	23 (8.2)	0.898	1.18 (0.10-13.92)
	10-12 years	11 (3.9)	0.81	1.36 (0.11-16.66)
	13-15 years	7 (2.5)	0.458	0.39 (0.03-4.77)
	16 years above	5 (1.8)	0.902	1.22 (0.05-27.44)
Educational level	Nursing diploma (institute)	42 (15.1)		
	Nursing diploma (College)	23 (8.2)	0.07	0.28 (0.07-1.11)
	Bachelor of Nursing	129 (46.2)	0.074	0.35 (0.11-1.11)
	Postgraduate in nursing	28 (10.0)	0.004	0.15 (0.04-0.55)

## Discussion

The findings of this study shed light on several important aspects of nursing staffing plans and the perceptions of nursing directors in government hospitals in Saudi Arabia [12].

The findings of this study align with existing literature highlighting the challenges of nursing staffing shortages in healthcare settings [13,14]. The acknowledgment of staffing gaps by nursing directors underscores the need for proactive workforce planning strategies to ensure adequate staffing levels and mitigate the potential negative impacts on patient care outcomes [15].

The preference for the professional judgment approach among nursing directors in estimating nursing staffing plans reflects the common reliance on subjective assessment rather than standardized methodologies. While professional judgment may offer flexibility, it can also introduce variability and subjectivity into staffing decisions. This highlights the importance of promoting evidence-based approaches and standardized tools for staffing calculations to enhance consistency and accuracy across healthcare facilities [16].

The findings also reveal variations in the utilization of different methods for estimating nursing staffing plans, with a majority favoring professional judgment over other standardized approaches [17]. This disparity may stem from factors such as familiarity, perceived ease of use, and organizational culture. However, promoting the adoption of evidence-based methods, such as patient acuity-based staffing models, could contribute to more effective resource allocation and improved patient outcomes [18].

The reliance on memos and special courses for training and knowledge enhancement among nursing directors underscores the importance of ongoing education and professional development initiatives [19]. This finding reinforces the need for accessible and tailored training programs to equip nursing leaders with the skills and knowledge required for effective staffing decision-making. Furthermore, the recognition of the need for standardized guidelines or standards for estimating nursing staffing plans underscores the importance of establishing clear benchmarks and best practices in workforce planning [4].

The association between nursing education level and staffing estimation approaches is consistent with previous studies demonstrating the influence of academic preparation on nursing practice. Nurses with higher levels of education may have a greater understanding of evidence-based practices and a propensity for critical thinking, which could influence their approach to staffing decisions [20]. This highlights the importance of investing in nursing education and professional development to cultivate a skilled workforce capable of navigating complex staffing challenges [21].

Socio-demographic factors such as gender and years of experience also play a role in shaping perceptions and attitudes toward staffing estimation methods. Female nurses and those with fewer years of experience as directors were more likely to perceive customs and traditions as obstacles to applying calculation methods [22]. This suggests the need for targeted interventions and support mechanisms to address barriers and promote evidence-based practices among nursing leaders.

Overall, this study contributes valuable insights into the complexities of nursing staffing plans in government hospitals in Saudi Arabia. By addressing staffing shortages and promoting evidence-based approaches to workforce planning, healthcare organizations can enhance the quality and safety of patient care delivery while optimizing resource utilization.

The present study has several limitations that should be acknowledged. Sampling bias may have occurred due to the cross-sectional design and the specific focus on nursing directors in government hospitals, which may not be representative of all healthcare settings in Saudi Arabia. The data collected through the survey questionnaire relies on self-reported responses from participants, which could be subject to recall bias or social desirability bias. Language and cultural factors may have affected the comprehension and interpretation of questions among participants, particularly if there were nuances in translation between English and Arabic. The findings from the study may not be generalizable to all healthcare settings in Saudi Arabia or to other countries with different healthcare systems and cultural contexts. Future research should consider addressing these limitations to provide a more comprehensive understanding of nursing staffing plans in Saudi Arabia.

## Conclusions

In conclusion, the study provides valuable insights into nursing shortages and estimation methods in Saudi Arabia's government hospitals. Addressing this challenge requires concerted efforts to enhance nurse leaders' knowledge and skills in staffing calculation methods. Standardized training programs, coupled with policy interventions, are essential for ensuring optimal nursing workforce planning and improving patient care outcomes.

## References

[1] Kane RL, Shamliyan T, Mueller C, Duval S, Wilt TJ (2007). Nurse staffing and quality of patient care. Evid Rep Technol Assess (Full Rep).

[2] Juvé-Udina ME, González-Samartino M, López-Jiménez MM (2020). Acuity, nurse staffing and workforce, missed care and patient outcomes: A cluster-unit-level descriptive comparison. J Nurs Manag.

[3] Twigg D, Duffield C (2009). A review of workload measures: a context for a new staffing methodology in Western Australia. Int J Nurs Stud.

[4] Griffiths P, Saville C, Ball J, Jones J, Pattison N, Monks T (2020). Nursing workload, nurse staffing methodologies and tools: A systematic scoping review and discussion. Int J Nurs Stud.

[5] Michel O, Garcia Manjon AJ, Pasquier J, Ortoleva Bucher C (2021). How do nurses spend their time? A time and motion analysis of nursing activities in an internal medicine unit. J Adv Nurs.

[6] Drennan VM, Ross F (2019). Global nurse shortages—the facts, the impact and action for change. Br Med Bull.

[7] Saville CE, Griffiths P, Ball JE, Monks T (2019). How many nurses do we need? A review and discussion of operational research techniques applied to nurse staffing. Int J Nurs Stud.

[8] George V, Massey L (2020). Proactive strategy to improve staff engagement. Nurse Lead.

[9] Al Asmri M, Almalki MJ, Fitzgerald G, Clark M (2020). The public health care system and primary care services in Saudi Arabia: a system in transition. East Mediterr Health J.

[10] Aboshaiqah A (2016). Strategies to address the nursing shortage in Saudi Arabia. Int Nurs Rev.

[11] Ball JE, Griffiths P (2022). Consensus Development Project (CDP): An overview of staffing for safe and effective nursing care. Nurs Open.

[12] Alluhaybi A, Usher K, Durkin J, Wilson A (2024). Clinical nurse managers’ leadership styles and staff nurses’ work engagement in Saudi Arabia: A cross-sectional study. PLoS One.

[13] Ross J (2002). A looming public health crisis: the nursing shortage of today. J Perianesth Nurs.

[14] Janiszewski Goodin H (2003). The nursing shortage in the United States of America: an integrative review of the literature. J Adv Nurs.

[15] Lu L, Ko YM, Chen HY, Chueh JW, Chen PY, Cooper CL (2022). Patient safety and staff well-being: organizational culture as a resource. Int J Environ Res Public Health.

[16] Engle RL, Mohr DC, Holmes SK, Seibert MN, Afable M, Leyson J, Meterko M (2019). Evidence-based practice and patient-centered care: Doing both well. Health Care Manage Rev.

[17] Griffiths P, Saville C, Ball JE, Jones J, Monks T (2021). Beyond ratios - flexible and resilient nurse staffing options to deliver cost-effective hospital care and address staff shortages: A simulation and economic modelling study. Int J Nurs Stud.

[18] Dall’Ora C, Saville C, Rubbo B, Turner L, Jones J, Griffiths P (2022). Nurse staffing levels and patient outcomes: A systematic review of longitudinal studies. Int J Nurs Stud.

[19] Mlambo M, Silén C, McGrath C (2021). Lifelong learning and nurses' continuing professional development, a metasynthesis of the literature. BMC Nurs.

[20] Ghodsi Astan P, Goli R, Hemmati Maslakpak M, Rasouli J, Alilu L (2022). The effect of evidence-based nursing education on nurses' clinical decision making: A randomized controlled trial. Health Sci Rep.

[21] Kristoffersson U, Mineur A, Heim S, Mandahl N, Mitelman F (1987). Normal high-resolution karyotypes in three patients with the Holt-Oram syndrome. Am J Med Genet.

[22] Mansour M, Darawad M, Mattukoyya R, Al-Anati A, Al-Madani M, Jamama A (2022). Socio-demographic predictors of structural empowerment among newly qualified nurses: Findings from an international survey. J Taibah Univ Med Sci.

